# Clinical Nutrition of Critically Ill Patients in the Context of the Latest ESPEN Guidelines

**DOI:** 10.3390/medicina55120770

**Published:** 2019-12-02

**Authors:** Aleksandra Gostyńska, Maciej Stawny, Katarzyna Dettlaff, Anna Jelińska

**Affiliations:** Department of Pharmaceutical Chemistry, Poznan University of Medical Sciences, Grunwaldzka 6, 60-780 Poznań, Poland; gostynska.aleksandra@spsk2.pl (A.G.); dettlaff@ump.edu.pl (K.D.); ajelinsk@ump.edu.pl (A.J.)

**Keywords:** parenteral nutrition, enteral nutrition, intensive care, immunomodulatory nutrition

## Abstract

The group of patients most frequently in need of nutritional support are intensive care patients. This year (i.e., 2019), new European Society for Clinical Nutrition and Metabolism (ESPEN) guidelines of clinical nutrition in intensive care were published, updating and gathering current knowledge on the subject of this group of patients. Planning the right nutritional intervention is often a challenging task involving the necessity of the choice of the enteral nutrition (EN) or parenteral nutrition (PN) route of administration, time of initiation, energy demand, amino acid content and demand as well as the use of immunomodulatory nutrition. The aim of this study was to specify and discuss the basic aspects of the clinical nutrition of critically ill patients recommended by ESPEN guidelines. Clinical nutrition in intensive care seems to be the best-studied type of nutritional intervention. However, meta-analyses and clinical studies comparing EN and PN and their impact on the prognosis of the intensive care patients showed ambiguous results. The nutritional interventions, starting with EN, should be initiated within 24–48 h whereas PN, if recommended, should be implemented within 3–7 days. The recommended method of calculation of the energy demand is indirect calorimetry, however, there are also validated equations used worldwide in everyday practice. The recommended protein intake in this group of patients and the results of insufficient or too high supply was addressed. In light of the concept of immunomodulatory nutrition, the use of appropriate amino acid solutions and lipid emulsion that can bring a positive effect on the modulation of the immune response was discussed.

## 1. Introduction

Clinical nutrition consists of providing all the nutrients necessary for the proper functioning of the body by the alternative to the oral administration route. Well planned and conducted nutritional intervention may improve the prognosis of the patients [1,2]. For critically ill patients, clinical nutrition is one of the basic elements of comprehensive therapy. Patients being admitted to intensive care units often due to the inability to oral intake, malnutrition, and coexisting catabolism resulting from the critical illness require the implementation of clinical nutrition. Its inclusion involves the supply of special preparations via artificial access to the gastrointestinal tract (enteral nutrition, EN) or intravenous infusion of a parenteral nutrition admixture (parenteral nutrition, PN). When planning the nutritional intervention, many aspects should be taken into consideration such as the time of initiation, energy demand, amino acid content and demand, and the use of immunomodulatory nutrition [3]. The EN is administered via nasogastric tube or gastrostomy/jejunostomy. The PN admixture contains all the necessary nutrients, i.e., amino acids, glucose, lipids, electrolytes, water, vitamins, and trace elements. The intravenous supply of such drugs requires adequate vascular access. This procedure is associated with an increased risk of infection and complications occurring during the obtaining of and maintaining access to the central vein and is therefore considered risky. Nevertheless, properly planned and conducted EN or PN nutritional intervention can improve prognosis, shorten the patient’s stay in hospital, and reduce treatment costs [4]. On the other hand, errors in nutritional intervention may lead to potential harm to patients’ health. This year (i.e., 2019), new European Society for Clinical Nutrition and Metabolism (ESPEN) guidelines of clinical nutrition in intensive care were published, updating and gathering current knowledge on the subject of this group of patients. The present study aimed to specify and discuss basic aspects of clinical nutrition in intensive care patients in the light of the latest recommendations of ESPEN [3]. Clinical nutrition consists of providing all the nutrients necessary for the proper functioning of the body by the alternative to the oral administration route.

## 2. Meta-Analyses and Clinical Studies Comparing PN and EN

The results of meta-analyses and clinical studies comparing the impact of the type of nutritional intervention on the safety and prognosis of patients differ depending on the chosen endpoints and study group [1,2,5,6,7,8,9]. The study performed on 2400 critically ill patients in 33 centers, comparing the 5 days of early nutritional support delivered via the parenteral and enteral route, showed no differences in mortality at 30 days, complication rate (infection), and period of hospitalization among patients admitted to intensive care units. The episodes of hypoglycemia and vomiting were significantly higher in the enteral group what can be explained by the impaired absorption of nutrients from gastrointestinal track and intolerance of enteral diets characterized by a quite high osmolality [6]. Critically ill patients often require additional medical interventions, such as the use of the respirator, the performance of dialysis, or the administration of drugs. The NUTRIREA-2 study included 2410 patients requiring mechanical ventilation and administration of vasopressors. The results did not show the advantage of EN over PN. However, it was shown that in patients in shock, the early isocaloric EN did not reduce the mortality or the risk of secondary infections, but was associated with the increased risk of gastrointestinal complications compared to the early isocaloric PN [7]. Both studies CALORIES and NUTRIREA-2 undertook the problem of nutritional support in critically ill patients, but there are some limitations that may alter the interpretation of the findings. The administration route of PN and EN cannot be masked thus both studies were nonblind, which could cause bias during data collection and interpretation. In the case of NUTRIREA-2 [7] the questioning issue is a nutritional protocol. Both study groups received full caloric nutrition EN or PN within the first 24 h after intubation. This procedure is not in accordance with the current standards, especially for patients not completely hemodynamically stable. Recent large RCTs, EDEN, suggest even that full caloric EN has some disadvantages over hypocaloric EN [10]. Furthermore, patients randomized to the early PN group after 3 days of PN administration could be switched to EN in the case of shock resolution, which also may affect the chosen endpoints. In the case of CALORIES [6], patients received lower doses of calories gradually increased to reach the caloric target by day 3. Furthermore, the exclusive PN or EN was conducted within 5 days. Changes in the nutritional intervention after this time period might alter the results concerning 30 days mortality. The meta-analysis included 18 randomized controlled trials studying 3347 patients showed that the use of EN as compared to PN has no effect on overall mortality but decreases infectious complications and length of stay in the intensive care unit. The authors noted that it was probably associated with reduced macronutrient intake than the enteral route itself [1]. Similar findings were shown by Zhang et al. [2] who additionally indicated that the use of EN was associated with increased risk of gastrointestinal complications. In contrast, the positive effect of EN supply in comparison with PN administration to intensive care patients was shown in other studies. Lewis et al. [8] analyzed 25 studies comparing EN vs. PN and EN vs. combination of EN and PN. Authors found fewer deaths at 30 days when studies gave combined EN and PN, and reduced sepsis for EN rather than PN. However, they concluded that data were insufficient to determine whether EN or combination of EN and PN is better or worse than PN, for mortality in hospital, at 90 days and at 180 days, and on the number of ventilator-free days and adverse events [8]. In another study concerning the assessment of the same nutritional interventions, authors showed that the administration of EN alone decreased the respiratory infections and length of days at the hospital for critically ill patients [9]. In the case of patients with severe pancreatitis, a positive impact of EN compared to PN was demonstrated. The meta-analysis of five clinical studies involving 348 patients showed that EN can help reduce the general mortality and the multi-organ failure rate and should be recommended as the preferred nutritional support for patients in this condition [5]. All mentioned meta-analyses have some limitations. Compared PN and EN interventions vary in reporting the caloric intake, the time of initiation and duration of nutrition intervention, and the definitions used for infections (when applied) as well. The number of enrolled patients in some included trials was limited (*n* < 100), significant heterogeneity of analysis of length of stay in ICU (intensive care unit) and differences in nutritional intervention weaken the strength of the conclusions. Therefore, we cannot be sure that the obtained results indicate the superiority of one administration route over the other result from the supply method itself, or other factors such as the qualitative and quantitative composition (e.g., protein intake) of the preparations used or the time of nutritional intervention. Except the results obtained in meta-analysis concerning patients with severe pancreatitis, where the study narrowed to the chosen subpopulation, the results of the other meta-analyses may not be applicable to a specific group of critically ill patients. Clinicians should choose the suitable nutritional administration route according to the actual clinical situation taking into account gastrointestinal function and metabolism rather than simply providing EN immediately or regarding the parenteral route as less safe. Despite the mentioned studies comparing early EN and PN in ICU patients, withholding early parenteral nutrition for 1 week, as suggested in the EPaNIC study [11], and conducting a trophic EN may be the best clinical option. The health care professionals should be aware of that the nutrition intervention may not bring improvement in clinical outcomes but cause potential harm to patients’ health.

## 3. Comparison of ESPEN Guidelines on Initiation of Clinical Nutrition for Intensive Care Patients and Other Patient Groups

Depending on the clinical situation, the guidelines of the European Society for Clinical Nutrition and Metabolism (ESPEN) specify the different recommended times of initiation of the nutritional intervention. In the case of critically ill patients and those with polimorbidities, the nutritional intervention, starting with EN, should be initiated within 24–48 h, if no oral nutrition is anticipated, satisfying 100% of the demand within 3 days [3,12]. If recommended, parenteral nutrition (PN) in critically ill patients should be implemented within 3–7 days [3], and within 7 days in surgical patients [13]. In the case of cancer patients, PN should be started when oral or enteral supply does not provide 60% of the energy demand within 10 days [4]. Similarly, in the case of geriatric patients, PN should be implemented, if it is anticipated that oral or enteral nutrition (EN) will be impossible for 3 days or will provide less than 50% of the demand for nutrients for more than 7 days [14]. It should be noted that both chemotherapy and radiotherapy are not contraindications to conduct EN and PN. The age of the patient is not a disqualification factor from the implementation of the nutritional intervention, although it is recommended to only use EN and PN in geriatric patients when there is a real chance to improve the health and/or the quality of life of the patient [14]. 

In the case of other disease entities, the indications for the initiation of the nutritional intervention depend on the health and state of nutrition before the occurrence of the disease. In mild pancreatitis, spontaneous recovery with the resumption of oral food intake usually occurs within 3–7 days, thus EN and PN are indicated only in malnourished patients or in those who are expected to have a longer time of inability to intake food orally. In the case of patients with pancreatitis and the indication for the initiation of PN, it is necessary to administer appropriate drugs for hemodynamic compensation, which usually takes place within 24–48 h [15]. 

Indications and contraindications for PN in the case of acute renal failure are comparable to the indications in other patients who are critically ill. In other kidney diseases, EN should be implemented in patients with high protein loss and intaking less than 20 kcal per kg of body weight per day [16]. Liver failure associated with alcoholism is the indication for the immediate implementation of PN if EN or oral nutrition is not enough. In the case of liver failure of a different cause, PN should be initiated when EN or oral nutrition cannot be used for more than 3 days [17].

Patients with short bowel syndrome often require PN for the first 7–10 days after the surgery. Depending on the location of the anastomosis and the length of the left part of the intestine, oral nutrition may be resumed or PN may be continued [18]. Regardless of the reasons for the initiation of PN, patients who do not have to continue hospitalization, and are indicated for PN, may be included in the Home Parenteral Nutrition (HPN) care. The only contraindication for the use of this type of procedure is the expected short period of patient survival [19].

So far, the time of initiation of PN in critically ill patients has been discussed. In previous ESPEN recommendations, it was indicated that the initiation of PN, as well as EN, should take place within 24–48 h [20]. In the randomized, multicenter EPaNIC study of 4640 critically ill adults, the early start of PN was compared (pursuant to the ESPEN guidelines published in 2009 [20]) to the late nutritional intervention (pursuant to the guidelines of American and Canadian associations [21,22]). Patients receiving late PN were less likely to develop infections, cholestasis, and the need for renal replacement therapy and mechanical ventilation. However, it should be noted that regardless of the nutrition scheme used, the death rate in the intensive care unit and the survival rate after 90 days were similar in both groups and did not differ statistically significantly [11]. The study protocol assumed in the case of insufficient enteral nutrition during the first 7 days after ICU admission in severely ill patients at risk for malnutrition, early or delayed supplemental parenteral nutrition administration. The early PN supply strategy revealed to be inferior to the late administration with the proviso that vitamins, trace elements, and minerals were provided to both groups of patients from the very beginning to avoid complications associated with the refeeding syndrome. Nevertheless, this study is not free from limitation. Firstly, the commercial PN admixtures administered to the patients during the study were characterized by a relatively low protein-to-energy ratio. Secondly, none of the patients received any of immune-modulating compounds (glutamine nor omega-3 acids), which may have a positive effect on the patients’ outcomes. Thirdly, some of the outcomes (such as duration of stay or functional status) were not determined by blinded assessors which also may lead to detection bias. Nerveless, in accordance with EPaNIC study [11], the latest ESPEN recommendation promote late rather than early PN intervention [3]. 

## 4. Energy Demand

In addition to the indications and the recommended time of the implementation of the nutritional intervention, the ESPEN guidelines also specify the demand for individual nutrients and the overall energy demand (Table 1). The studies concerning PN made it possible to better understand the biochemical and metabolic aspects related to the supply of nutrients. The recommended method of calculation of the energy demand is indirect calorimetry. This method, giving a true picture of the patient’s energy expenditure, allows for the preparation of an individualized diet. Indirect calorimetry had become indispensable in pediatric intensive care units [23]. In critically ill adults, half of the patients in intensive care units had indications for the measurements of energy expenditure using indirect calorimetry [24]. The assessment of energy expenditure in patients requires indirect calorimetry and cannot be predicted by equations due to the acute or chronic conditions affecting the metabolic characteristics, reflected by highly variable EE (energy expenditure) [25]. Another important factor preventing the use of predictive equations based on simple anthropometric measures is body composition, which makes it a challenge to calculate energy expenditure in patients overfeeding or underfeeding [26]. Tatucu-Babet et al. in a systematic review demonstrated that in general the predictive equations yielded an acceptable estimation for only half of the intensive care patient population while the rest were either over- or underestimated [27].

Nevertheless, due to the complexity and considerable cost of indirect calorimetry, it is still not used routinely. In everyday practice, various mathematical formulas are used to estimate the energy demand and the need for individual nutrients. The basis for the calculation of the energy demand is the Harris–Benedict equation, developed at the beginning of the 20th century [28]. Nowadays, it is believed that only 50% of the healthy population has the basic energy expenditure of approximately ±10% to the value obtained from the Harris–Benedict equation, and this discrepancy is often much larger in critically ill patients.

Predictive equations were developed on the basis of population studies of a specific group of people and applying them to an individual may lead to the occurrence of a significant error, especially if the individual does not share important characteristics with the studied group. For this reason, besides the equation developed by Harris and Benedict, there were many other predictive equations developed to meet the acceptance estimation for different groups of people. Three of them were identified as commonly used and thus clinically important: equations developed by Owen [29,30], World Health Organization/Food and Agriculture Organization/United Nations University WHO/FAO/UNU [31], and Mifflin–St Jeor [32,33]. Among those equations, the Mifflin–St Jeor equation performed the best, characterized by the lowest errors (82% of the cases the prediction was 10% measured resting metabolic rate) in the energy expenditure determination [34]. 

In the guidelines concerning intensive care patients, Singer et al. [3] stated that if calorimetry is not available, using oxygen consumption from pulmonary arterial catheter or carbon dioxide production derived from the ventilator will give a better evaluation of energy expenditure than predictive equations [3]. In the case of the use of the mathematical equation to estimate the energy needs, the hypocaloric nutrition (<70% of the calculated energy expenditure) should be implemented and the supply during the first 7 days should be increased [3].

## 5. Amino Acids Composition and Demand

Other questionable issues are the optimal dose and the composition of the amino acids for critically ill patients. From the very beginning of the use of PN, in the 1960s, there were discussions on the method of reduction of the catabolism in critically ill patients. The concept of hyperalimentation was then developed. This term suggested that malnourished patients or those with increased catabolism should receive more amino acids on purpose than their normal need for nutrients [35,36]. Properly selected (rich in protein and energy) PN, according to the concept of hyperalimentation, was supposed to prevent the catabolism in critically ill patients. However, according to later studies, the supply of too large amounts of protein can cause harm rather than good. 

The ESPEN guidelines [3] concerning the protein demand among critically ill patients indicate that the amount of protein supplied should be 1.3 g per kg of the ideal body weight per day (Table 1). It is worth noting the difference between the perfect and the actual bodyweight that underlines the calculation of the protein demand. In critically ill patients, this difference can be significant due to fluid retention, positive fluid balance, and excess body fat.

In critically ill patients, muscular dystrophy and disturbances in the amino acids, glucose and lipid balance are very often. It is believed that stress factors accompanying critically ill patients cause the catabolism of muscle proteins, which give the possibility of prolonged gluconeogenesis and synthesis of acute-phase proteins in the state of insufficient supply of amino acids [36,37]. The considerable use of amino acids by non-muscle tissue may lead to hypoaminoacidemia [38], which in consequence, with prolonged stress, leads to muscle atrophy and degeneration, as well as significant loss of lean body mass [39]. 

It is also suggested that the increased protein demand is associated with the increased synthesis of cysteine, the amino acid reducing the rate of glutathione synthesis. This mechanism indirectly leads to the inhibition of oxidative stress and prevents the deficiency of glutamine in muscle and plasma [40,41,42]. Too low protein supply results in the reduced level of plasma proteins (albumin, prealbumin, transferrin, transport proteins), decreased muscle mass, impaired function of internal organs, and reduced immune response. In addition, it is noted that some of the endogenous amino acids, due to altered metabolism in critically ill patients, may become conditionally essential amino acids [41]. For this reason, when starting the nutritional intervention in critically ill patients, attention should be given not only to the amount of amino acids supplied but also to their composition and proportions. The ESPEN guidelines concerning the supply of amino acids to critically ill patients suggest a dose of 1.3 g per kg of the ideal body weight per day [3]. Such a dose is justified by the increased catabolism and increased protein demand. In the meta-analysis concerning nitrogen balance studies in 1107 patients, Kreymann et al. [43] noted that proteolysis (measured with the concentration of nitrogen in urine) is significantly increased in critically ill patients and exponentially associated with the severity of the patient’s clinical condition. Wolfe et al. [44] and Shaw et al. [45] demonstrated that in patients with serious injuries, receiving the appropriate amount of calories, regardless of the type of nutrition (EN or PN), about one-third of the amino acids supplied is used for the protein synthesis (anabolism), and two-thirds are subject to catabolism. However, it should be noted that too high protein supply may also lead to dangerous complications as a result of increased ureagenesis. 

The Nephro-Protective study showed that the administration of high doses of amino acids (2.0 g per kg of body weight per day) correlated with the increased demand for renal replacement therapy (RRT) [46]. This study is the first multicenter randomized controlled trial to show a physiological effect of a nutritional intervention the renal function express, among others, as a glomerular filtration rate estimated (eGFR) and urinary output in critically ill patients. Regarding the result indicating the trend for the need for RRT in patients receiving amino acid infusion, the question is whether the requirement for RRT was really influenced by the PN administration and was really needed. This intervention tended to be initiated when the higher blood urea nitrogen appeared. At the initiation of RRT other parameters of kidney function e.g., urine output, serum creatinine concentration did not differ between groups or they were even better in the treatment group compared to the reference group. Increased ureagenesis, however, disproportionate to changes in creatinine concentrations in plasma, probably indicates the catabolism of amino acids supplied with PN. Critically ill patients also have an elevated concentration of glucagon, a catabolic hormone responsible, among others, for the catabolism of amino acids. The increased level of glucagon in plasma is responsible for the decomposition of amino acids in the liver. This phenomenon is additionally intensified by the infusion of amino acids and does not protect against the degradation of muscle proteins [47]. In addition, the supply of increased amounts of protein leads to thermogenesis, meaning a significant increase in body temperature, often misinterpreted as a sign of infection and treated with antibiotics. The nutrient supply, significantly exceeding the demand, intensifies the energy expenditure. The diet-induced thermogenesis (DIT) is particularly unfavorable in critically ill patients with limited respiratory or cardiovascular system efficiency [48]. The DIT depends on the energy source being administered and is the highest in the case of protein and amino acids (20%–30% of the energy supplied from proteins and amino acids is converted into thermal energy). The remaining nutrients are characterized by the DIT with significantly lower values (5%–10% for carbohydrates and 0%–3% for fat emulsions) [49]. Research on a misinterpretation of the thermal effect of high protein supply is limited, which may indicate that this scenario is rarely seen. However, understanding the mechanism of the thermal effect of intravenous amino acid supply allowed their use in patients undergoing anesthesia to suppress hypothermia [50].

Ferrie et al. [51] studied the group of 120 critically ill patients requiring PN, comparing the effects after the administration of a high dose (ultimately 1.2 g per kg of body weight per day) and a low dose of amino acids (ultimately 0.8 g per kg of body weight per day). The primary end point in the study was the grip strength of patients, which did not differ statistically significantly in both groups. The grip strength of patients receiving higher doses of amino acids was stronger on the 7th day of the study. However, both short-term and long-term (6-month) mortality in this group of patients was numerically higher, although not statistically insignificant. These results show that the supply of lower doses of amino acids than recommended may not have a negative impact on the prognosis of critically ill patients. However, it should be noted that patients included in the study, in addition to the different amino acid supplies, received various doses of glucose (higher in patients receiving fewer amino acids), which could affect the final results of the study [51]. Despite numerous reports supporting the current recommendations of scientific associations concerning the supply of amino acids in critically ill patients, there is still a discussion questioning the benefits of early amino acids supplementation which, according to some authors, may lead to the inhibition of autophagy and the catabolic process by supplied amino acids [52]. The inhibition of autophagy was observed in both animal models and critically ill patients [53,54]. Moreover, it has been assumed for a long time that macronutrients, in particular, amino acids, inhibit the hypercatabolic body response to serious injury and stress, considered to be desirable. The degradation of muscle proteins, observed in critically ill patients, is considered an adaptation process, aimed at the creation of substrates necessary for gluconeogenesis and supply of glucose to the most important organs and systems from the point of view of the body survival [41]. It is believed that the administration of exogenous amino acids inhibits the process of release of essential amino acids and stimulates the synthesis of muscle proteins, and after exceeding the anabolic capacity of the body, they are transformed to urea in the liver, increasing ureagenesis, which may lead to both liver and kidney damage [55,56]. 

Concluding the presented discussion of literature concerning the choice of appropriate amino acid dose, we state that the administration of the amino acid in the dose calculated according to well-established ESPEN guidelines [3] should bring the most benefits for patients as it was shown that both too high and too low doses of amino acids may worsen patient outcomes.

## 6. Immunomodulatory Nutrition

The traditional approach to clinical nutrition consisted of the supply of nutrients in order to maintain lean body mass and migrate the effects of catabolic processes, i.e., to minimize the negative energy and nitrogen balance, as well as to maintain the proper function of organs and systems. The contemporary concept of nutritional therapy raises the issue of not only maintaining lean body mass and the nutritional condition of the patient, but also the effect of nutrition on the modulation of the immune response, alleviation of the metabolic response to stress, the reduction of oxidative cell damage, and the impact on the wound healing process. The use of ingredients that may have properties modulating the immune system, such as glutamine, arginine, nucleotides, omega-3 fatty acids, gamma-linolenic acid, L-carnitine, and taurine, can substantially affect the healing process of the patient [57]. However, it should be noted that there are no large multicenter randomized studies confirming the positive effect of these substances in individual disease entities. At the same time, their negative effects have not been confirmed, thus they are increasingly used in patients. 

The administration of preparations enriched with selected amino acids may be justified, e.g., in patients with serious injury who have reduced levels of glutamine, arginine, citrulline, and taurine in blood [40,58,59]. The supplementation of parenteral nutrition with these amino acids in higher than standard doses may improve treatment outcomes. Until now, PN glutamine supplementation has been used in clinical practice. Glutamine, a conditionally essential amino acid, is a precursor of nucleotide synthesis and a hepatic gluconeogenesis substrate. It is an important energy source for rapidly dividing and renewing cells, which is why it has a positive effect on the gastrointestinal epithelium and lymphocytes. Glutamine deficiency can occur under catabolic stress conditions when, as a result of its increased metabolism and despite the intense release from skeletal muscles, there are significant decreases in its concentration in the blood. Organs particularly exposed to glutamine deficiency include the liver, digestive tract, and kidneys. The use of glutamine in cancer patients may positively affect the functioning of the immune system, however, in some cases, such as breast cancer, it should be avoided. In-vitro studies demonstrated the negative effect of glutamine on some breast cancer lines [60]. Glutamine deficiency contributes to the dysfunction of the immune system and increased mortality [40,61]. Meta-analyses of randomized studies suggest that glutamine and antioxidant supplementation in critically ill patients may be associated with prolonged survival [62,63]. At the same time, however, the clinical study performed on the group of 1223 critically ill patients showed that the administration of glutamine compared to placebo did not have any clinical benefits, and on the contrary, it could be associated with increased mortality [64]. In this study, the early provision of glutamine in the dose of 0.35 g per kg of body weight per day intravenously and 30 g enterally plus antioxidants was associated with increased mortality at 28 days among critically ill patients with multiorgan failure as compared with those who receive standard clinical nutrition support [64]. The chosen dose of glutamine seems to be high and could have had an impact on the results of the study, by leading to an amino acid imbalance. Providing inadequate or imbalance composition of amino acid may have colossal clinical consequences, for instance, the use of essential amino acids only and/or eliminating the arginine form the parenteral diets had been shown to produce hyperammonemia [65,66]. Therefore, further clinical studies appear to be necessary to determine safe doses of glutamine for critically ill patients especially with multiorgan failure. Wischmeyer, on the basis of recent meta-analyses, suggested that traditional glutamine supplemented PN admixtures is safe and should be provided in patients in need of PN, with burns, trauma, or malignancies in balanced doses at less than 0.35 g/kg/day (parenterally) or at less than 0.5 g/kg/day (enterally) [67]. However, the ESPEN recommendations [3] stated that in the case of unstable and complex critically ill patients, particularly in those suffering from liver and renal failure, parenteral administration of glutamine should be avoided. The enteral administration is recommended in patients with burns >20% body surface area (0.3–0.5 g per kg body weight per day for 10–15 days as soon as EN is commenced), in critically ill trauma (0.2–0.3 g per kg body weight per day for 5 days), and in the case of complicated wound healing (0.2–0.3 g per kg body weight per day for 10–15 days) [3].

Patients with extensive postoperative wounds or wounds resulting from the injury may require the administration of arginine enriched diets. It is a conditionally essential amino acid that plays an important role in nitrogen transformations, and thus in protein synthesis. Arginine participates in the synthesis of nitrogen oxide and polyamines and stimulates the secretion of growth hormone, glucagon, insulin, prolactin, and somatostatin. Its absence in the diet impairs the protein synthesis and prolongs the wound healing process [59]. 

Immunomodulatory diets can also be enriched with nucleotides. These compounds participate in almost all biochemical processes, are the energy source in the cell, regulate metabolism, and mediate many metabolic pathways. The states of increased demand for nucleotides are a heavily surgical procedure, injury, or septic syndrome. In these situations, there is a significantly increased catabolism of nucleotides that exceeds their de novo synthesis and resynthesis. Fast-proliferating tissues, such as intestinal epithelium, cells of the immune and hematopoietic system are particularly exposed to nucleotide deficiencies. It is believed that the supplementation with nucleotides accelerates the regeneration of intestinal villi and improves the function of the immune system [67,68].

The use of lipid emulsions, containing appropriate polyunsaturated fatty acids, may lead to prolonged survival and shortened hospitalization [3,69,70]. The latest ESPEN recommendation stated that PN and EN enriched in omega-3 acids can be administered to critically ill patients [4]. The source of eicosapentaenoic acid (EPA) and docosahexaenoic acid (DHA), both in PN and EN, is fish oil rich in omega-3 fatty acids. The precursor of omega-3 acids is α-linolenic acid (ALA), but it does not participate in the modification of inflammatory responses. This is due to the very low affinity of this acid for Δ6-desaturase, the enzyme converting ALA into biologically active fatty acids (EPA and DHA). EPA and DHA are transformed into anti-inflammatory prostaglandins, leukotrienes, resolvins, protectins, and maresins that compensate for proinflammatory mediators arising from arachidonic acid. In addition, the positive modulation of the inflammatory process also involves the incorporation of EPA and DHA into cell membranes, which causes a decrease in the concentration of arachidonic acid in phospholipid membranes and, as a consequence, the reduction in the production of pro-inflammatory eicosanoids [69,70]. The authors of the latest review concerning the impact of intravenous lipid emulsions containing fish oil on clinical outcomes in critically ill surgical patients demonstrated that those lipid emulsions positively affect the functioning of the body by lowering triglyceride concentrations, inflammatory markers, and liver function enzymes, improving patients morbidity and mortality [71].

## 7. Conclusions

Nutrition of critically ill patients is an indispensable element of holistic intensive care. Proper planning, taking into account the appropriate route of administration, as well as estimating energy and nutritional needs can significantly improve the patient’s prognosis and outcomes. The use of indirect calorimetry is the gold standard for determining patients’ energy expenditure. However, due to the limited access to this type of apparatus, there are predictive equations in clinical practice. The latest ESPEN guidelines indicate that clinical nutrition should be considered for each patient remaining in the intensive care unit for more than 48 h. If the oral food intake is not possible the EN should be implemented within 48 h. In the case of contraindications to EN, PN should be implemented within 3–7 days. The recommended dose of amino acid is 1.3 g per kg bodyweight. The use of immunomodulatory nutrition in intensive therapy is debatable, but it seems that the advantages of this type of preparation outweigh the risk of their use.

## Figures and Tables

**Table 1:** European Society for Clinical Nutrition and Metabolism (ESPEN) guidelines for intensive care in comparison to other ESPEN recommendations [8,9,10,11,12,13,14,15,16,17].

ESPEN Guidelines	Indications/Initiation of Clinical Nutrition	Daily Energy Demand	Daily Amino Acids Demand	Daily Glucose Demand	Daily Lipids Demand
Intensive care Singer et al., 2019 [3]	Clinical nutrition should be considered for each patient remaining in the ICU for more than 48 h. EN must be implemented within 48 h, if oral intake is not possible. PN should be implemented within 3–7 days in the case of contraindications to EN.	20–25 kcal/kg bw	1.3 g/kg bw	Max. 5.0 mg/kg bw/min	Max. 1.5 g/kg bw
Surgery Braga et al., 2009 [13]	PN should be implemented in patients who are unable to receive and/or absorb diets administered orally or enterally for at least 7 days.	25 kcal/kg ideal bw; in conditions of severe stress 30 kcal/kg ideal bw	1.5 g/kg ideal bw or about 20% of EE	The energy ratio: amino acids:glucose:lipids 20:30:50 or glucose:lipids 50:50, 60:40, or 70:30	
Gastroenterology Van Gossum et al., 2009 [18]	Clinical nutrition is necessary for the first 7–10 days after surgery.	0.85–1.5 × REE 25–33 kcal/kg bw	1.0–1.5 g/kg bw	No recommendation	Max. 1.0 g/kg bw
							The energy ratio: glucose:lipids 66:33	
Non-surgical oncology Bozzetti et al., 2009 [4]	Short-term PN is usually required. PN should be implemented when oral or enteral administration does not ensure min. 60% of energy demand in 10 days.	20–25 kcal/kg bw for inpatients; 25–30 kcal/kg bw for outpatients	No recommendation The most commonly used doses are in the range of 1.0 g/kg bw to 1.2–2.0 g/kg bw	No recommendation Patients with insulin resistance The energy ratio: glucose:lipids 50:50	
Geriatrics Volkert et al., 2018 [14]	If EN is indicated, it should be implemented as soon as possible. PN should be implemented if it is anticipated that oral and EN administration will be impossible for more than 3 days or <50% of supply over 7 days.	30 kcal/kg bw Oral nutritional support should provide min. 400 kcal including min. 30 g of protein	Min. 1.0 g/kg bw	No recommendation	
Polymorbid internal medicine patients Gomes et al., 2017 [12]	EN or PN should be implemented within 48 h.	EE 27 kcal/kg actual bw (>65 years) REE 18–20 kcal/kg bw REE 30 kcal/kg bw in severe malnutrition	Min. 1.0 g/kg bw	No recommendation	
HPN Staun et al., 2009 [19]	HPN is indicated in patients who can stay at home and who are unable to receive and/or absorb diets administered orally or enterally and there is a risk of death due to malnutrition. HPN is not recommended for patients with the expected short period of survival.	20–35 kcal/kg bw	0.8–1.0 g/kg bw	Max. 7.0 mg/kg bw/min	1.0 g/kg bw in HPN >6 months
				100–150 kcal non-protein energy/g of nitrogen The energy ratio: glucose:lipids 60:40	
Hepatology Plauth et al., 2009 [17]	PN should be implemented if oral or EN administration is not possible for more than 3 days. In patients with alcoholic liver disease, PN should be implemented without delay if oral nutrition or EN is not sufficient.	1.3 × REE	1.2–1.5 g/kg bw 0.8–1.2 g/kg bw in acute liver failure	2.0–3.0 g/kg bw	0.8–1.2 g/kg bw
				50%–60% of non-protein energy in patients with alcoholic liver disease	
Renal failure Cano et al., 2009 [16]	In acute renal failure, PN is indicated if oral or EN nutrition is not possible. EN should be implemented in patients showing high protein loss and who intake less than 20 kcal/kg bw/day.	30–40 kcal/kg bw 30–35 kcal/kg bw in patients with chronic renal failure	1.1–1.5 g/kg bw	No recommendation	
Pancreatitis Gianotti et al., 2009 [15]	EN and PN are indicated in malnourished patients or when the period of famine is anticipated for more than 5–7 days. If EN is indicated, they should be implemented as soon as possible. PN should be implemented only when EN is impossible.	Nonprotein energy: 25–30 kcal/kg bw	No recommendation Note: parenteral amino acids do not affect the function and secretion of the pancreatitis.	No recommendation Note: parenteral carbohydrates do not affect the function and secretion of the pancreas.	0.8–1.5 g/kg bw

bw—body weight; EE—Energy Expenditure; REE—Resting Energy Expenditure.
